# Surgery After Microcoil Embolization in a Patient With Large Bilateral Intrathoracic Extramedullary Hematopoiesis and Thalassemia

**DOI:** 10.1016/j.atssr.2025.11.031

**Published:** 2025-12-24

**Authors:** Eric Francis Macharia-Nimietz, Jan-Christoph Zeisel, Martin Thanh-Long Takes, Didier Lardinois

**Affiliations:** 1 Department of Thoracic Surgery, University Hospital Basel, Basel, Switzerland; 2 Clinic for Vascular and Thoracic Surgery, Cantonal Hospital Baselland, Liestal, Switzerland; 3 Medical Radiological Institute Zürich, Zürich, Switzerland

## Abstract

We report the case of a 54-year-old woman with thalassemia intermedia and large bilateral intrathoracic extramedullary hematopoiesis since childhood. The patient presented with severe dyspnea and a suspected left-sided hemothorax on a computed tomography scan accompanied by almost complete atelectasis of the left lower lobe 3 weeks after blunt trauma. Microcoil embolization of the affected intercostal arteries was performed before a challenging but successful surgical resection.

Extramedullary hematopoiesis (EMH), the production of blood cells outside the bone marrow, occurs as a compensatory response in diseases like thalassemia, myelofibrosis, and hereditary spherocytosis. Common sites include the spleen and liver. Intrathoracic EMH is rare, typically located in the posteroinferior paraspinal area, and is usually asymptomatic, often not requiring surgery. Because of the hypervascularization, thoracic trauma can cause massive bleeding.^1^ We report the case of a patient with a hemothorax and nearly complete atelectasis of the left lower lobe treated with a hybrid approach involving preoperative embolization of the affected intercostal arteries with microcoils followed by surgery.

A 54-year-old woman presented with chronic lumbar spine pain. After falling from her bicycle, she visited the emergency department, where she was diagnosed with a lower leg fracture, a sprained ankle, and a knee contusion. Treatment with anti-inflammatory drugs, rivaroxaban (Xarelto; Bayer AG) for thromboprophylaxis, and plaster immobilization of the fracture was initiated, and she was discharged the same day. Three weeks later, she was referred to our institution because of severe dyspnea. A chest radiograph and computed tomography scan showed a left-sided pleural effusion with suspected hemothorax and nearly total atelectasis of the lower left lobe (Figures 1A, 1B).

**Figure 1:**
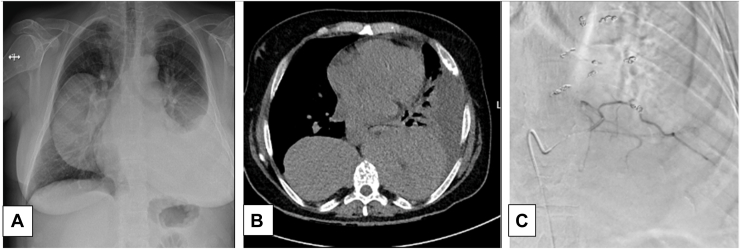
(A) Chest radiograph showing left-sided hemothorax and bilateral extramedullary hematopoiesis masses. (B) Computed tomography scan of the thorax showing the bilateral extramedullary hematopoiesis masses and the left-sided hemothorax. (C) Angiography image during embolization of the intercostal arteries with microcoils.

Laboratory findings revealed a hemoglobin level of 88 g/L (baseline, 119 g/L; normal, 123-153 g/L) and a hematocrit of 29%. The patient’s vital signs were normal, except for a slightly elevated pulse of 105 beats/min. We assessed standard treatment options, including observation, drainage, minimally invasive treatment, and surgery. The option of drainage alone was considered, but concerns arose about the large size of the EMH and the potential risk of injuring it, which could worsen the bleeding. Anemia from resection of the bleeding EMH was not a major concern as the contralateral EMH would maintain hematopoiesis. Given the risk of further bleeding from the well-vascularized mass potentially enlarging the suspected hemothorax under rivaroxaban therapy and the disabling chronic pain, we proposed a surgical exploration with the patient’s full consent.

We interpreted the tachycardia in association with the anemia as an early sign of hypovolemic shock. Therefore, she received a transfusion of 2 blood units before surgery. Interventional radiologists performed angiography with bilateral coil embolization (Tornado; Cook Medical) under fluoroscopic guidance of the intercostal arteries T9 to T11 on the left side and T10 and T11 on the right side (Figure 1C). The main intercostal artery was then entered with a 2.7F microcatheter (Progreat; Terumo), through which the embolization of the intercostal arteries with microcoils (2 mm/2 cm Hilal; Cook Medical) was performed by a front- and back-door approach (ie, proximal and distal to the lesion on the artery to prevent backflow of blood). Angiographic mapping was performed before embolization to clearly identify the artery of Adamkiewicz and other radiculomedullary arteries, which should be preserved to avoid spinal cord morbidity such as ischemia or infarction and chest wall ischemia or necrosis. The artery of Adamkiewicz was visualized arising from the left T12 and supplying the anterior spinal artery; therefore, no embolization was done there. The intercostal arteries T10 and T11 on the right side were embolized to prevent collateral flow and as prophylaxis against future bleeding.

The operation through thoracotomy confirmed the hemothorax. An extrapleural approach was chosen for resection because of persistent diffuse bleeding from the EMH despite precoiling. Achieving complete hemostasis was challenging, even with the use of argon beaming, clipping of small vessels, and compression of the bleeding sites. It was then decided to resect the entire mass at the level of the endothoracic fascia as leaving part of it posed a risk for further bleeding (Figure 2A). After resection, the tumor bed was extensively compressed. Floseal granulate (Baxter) and polyglycolic acid sheets (Neoveil; M Gunze Ltd) were applied, and complete hemostasis was finally achieved. The right-sided EMH was left in situ to avoid exacerbating anemia, with no plan for surgery on that side.

**Figure 2:**
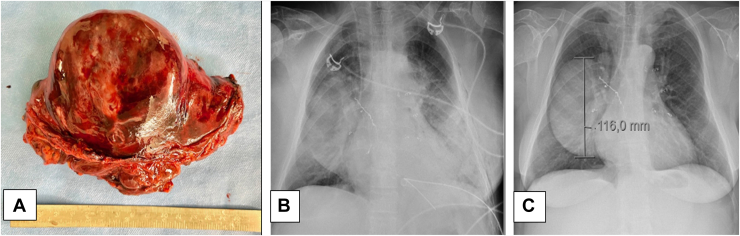
(A) Postoperative specimen of the left-sided extramedullary hematopoiesis after resection with the characteristic fatty appearance. (B) Postoperative chest radiograph. (C) Chest radiograph 4 weeks after the operation.

Chest radiographs from the postoperative day and 4 weeks later (Figures 2B, 2C) showed a well reexpanded lung without pleural effusion. The EMH was confirmed histologically. Postoperative recovery was uneventful, and the patient was discharged 15 days after the operation without any dyspnea. There were no signs of recurrence at the 1-year follow-up, and the hemoglobin levels remained stable. Although the patient reported reduced pain at rest compared with before surgery, she complained of intensified pain during activity. It is not clear why.

## Comment

In most cases, EMH remains asymptomatic. Diagnosis can be made by several noninvasive diagnostic techniques, including technetium Tc 99m sulfur colloid radionuclide bone marrow scanning, magnetic resonance imaging, contrast-enhanced computed tomography, and chest radiography. Radiographs typically show unilateral or bilateral, posterior or lateral, well-circumscribed lobulated paravertebral mass lesions, usually located caudal to the sixth thoracic vertebrae, without bone erosion or calcification.^1^

Surgical resection may even be harmful as it removes a compensatory source of erythropoiesis, potentially exacerbating underlying anemia.^1^ Radiotherapy can be used as an alternative since hematopoietic cells are radiosensitive^1^; however, its effects are too delayed to be used in acute situations. Surgery has been described for cases of hemothorax associated with EMH^1^, ^2^, ^3^ and is recommended only to control significant bleeding and to relieve compression of the spinal cord or other vital structures.^2^^,^^3^ Andras and colleagues^4^ found that the esophageal plexus and intercostal arteries supplied blood, whereas the venous drainage occurred through the azygos system. Embolization of the intercostal arteries with microcoils to prevent uncontrolled intraoperative bleeding is the common form of transcatheter embolization in managing traumatic hemothorax.^3^ Morbidities associated with coiling an entire intercostal artery over multiple levels are rare because of precoiling angiographic mapping that clearly identifies the artery of Adamkiewicz and other radiculomedullary arteries that should be preserved. These morbidities are more commonly associated with the use of particle or liquid embolizing agents for intercostal artery embolization. In our case, we decided to resect the lesion because of the risk of further uncontrolled bleeding and the patient’s disabling chronic pain. Even after embolization, surgery remains challenging. It is unclear whether the esophageal vascular plexus contributed to the continued oozing during operation as this was not embolized.

The patient was prescribed rivaroxaban for prophylaxis of deep venous thrombosis (DVT) after her injury. This is generally not recommended for patients with isolated lower limb injuries and low DVT risk. However, for our patient with thalassemia, a risk factor for DVT, prophylaxis was recommended.

In conclusion, surgical resection of EMH is feasible but challenging and requires an interdisciplinary approach. Precise angiographic mapping of the blood supply to the lesion should be considered preoperatively to identify any contributions from the esophageal vascular plexus. This approach can help avoid persistent oozing during surgery. This mapping also identifies spinal feeders that should be preserved during embolization to avoid complications like spinal cord ischemia and chest wall necrosis. Surgical resection should be performed by experienced surgeons.
